# Unusual enantiomeric D,L-*N*-acyl homoserine lactones in *Pectobacterium atrosepticum* and *Pseudomonas aeruginosa*

**DOI:** 10.1371/journal.pone.0283657

**Published:** 2023-03-31

**Authors:** Umang Dhaubhadel, Abiud E. Portillo, Ondřej Horáček, Yu-Sheng Sung, Daniel W. Armstrong

**Affiliations:** 1 Department of Chemistry & Biochemistry, The University of Texas at Arlington, Arlington, Texas, United States of America; 2 Department of Pharmaceutical Chemistry and Pharmaceutical Analysis, Faculty of Pharmacy, Charles University, Hradec Kralove, Czech Republic; Yenepoya University, INDIA

## Abstract

Quorum Sensing allows bacteria to sense their population density via diffusible *N*-acyl homoserine lactone (N-HL) signaling molecules. Upon reaching a high enough cell density, bacteria will collectively exhibit a phenotype. Until recently, methods used for detection of N-HLs have not considered the chirality of these molecules and it was assumed that only the L-enantiomer was produced by bacteria. The production and effects of D-N-HLs have rarely been studied. In this work, the temporal production of D-N-HLs by the plant pathogen *Pectobacterium atrosepticum* and the human pathogen *Pseudomonas aeruginosa* are reported. Both bacteria produced D-N-HLs in significant amounts and in some cases their concentrations were higher than other low abundance L-N-HLs. Previously unreported D-enantiomers of *N*-3-oxoacyl and *N*-3-hydroxyacyl homoserine lactones were detected in *P*. *atrosepticum*. Interestingly, L-N-HLs produced in the lowest concentrations had relatively higher amounts of their corresponding D-enantiomers. Potential sources of D-N-HLs and their significance are considered.

## Introduction

**Fig 1:**
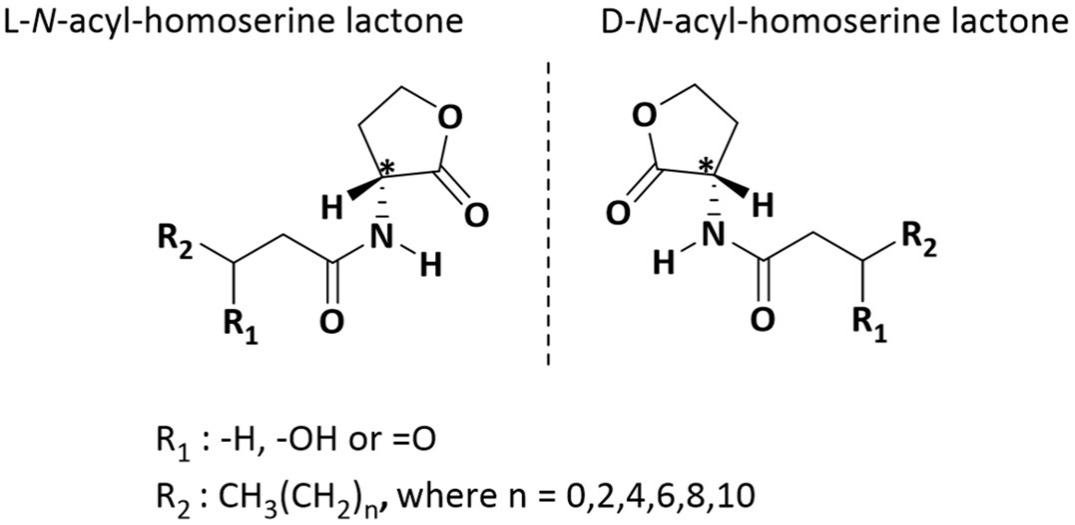
Structure of D,L-*N*-acyl homoserine lactones. R_1_ represents the substitution on the third position of the acyl chain and R_2_ represents the length of the acyl chain.

Bacterial cell-to-cell communication occurs via diffusible molecules in a process known as quorum sensing (QS) [1–3]. QS involves the biosynthesis of autoinducer molecules and their release outside the cell. Upon reaching a critical concentration, the molecules diffuse back into the cell and are responsible for regulating phenotypic expression. This phenomenon enables bacteria to reach a certain population before exhibiting a phenotype. *N*-acyl homoserine lactones (N-HLs) are the autoinducer molecules that are responsible for QS in gram-negative bacteria [1, 2, 4, 5]. N-HL molecules consist of a fatty acyl chain attached via an amide bond to a γ-butyrolactone ring, which contains a stereogenic center α to the carbonyl group of the lactone ring [6]. Diversity of N-HLs comes from the varying lengths of the acyl chains and the functionality of the acyl chain. As shown in Fig 1, the chain lengths can vary from 4 to 18 carbons and can be substituted with either a hydroxy or a carbonyl group at the third position of the acyl chain [7]. There is a second chiral center present on the acyl chain of the *N*-3-hydroxyacyl homoserine lactones. This results in two pairs of enantiomers [6, 8]. Different bacteria have been shown to have specific types of N-HLs to facilitate QS [6, 9].

This study involves two important bacteria: a plant pathogen *Pectobacterium atrosepticum* and the human pathogen *Pseudomonas aeruginosa*. Both bacteria have been shown to have QS mediated virulence factors. *P*. *atrosepticum* causes soft rot in potato tubers and potato blackleg disease [10, 11]. QS regulated by *N*-3-oxohexanoyl homoserine lactone in *P*. *atrosepticum* is responsible for producing various exoenzymes that cause plant cell wall degradation [12, 13]. *P*. *aeruginosa* has been shown to have distinct regulons for its two prominent N-HLs: *N*-butanoyl homoserine lactone and *N*-3-oxododecanoyl homoserine lactone. These N-HLs are responsible for triggering virulence factors that aid in biofilm formation, acute infections, and host cell damage [5, 14–16]. *P*. *aeruginosa* infections are prevalent in cystic fibrosis patients, and many of its strains are resistant to various antibiotics [16–18]. The connection between virulence factors and QS provides an important pathway to target these pathogens. N-HL degrading bacteria have been used to quench the quorum, and effectively reduce virulence factors of *P*. *atrosepticum* [19–21]. Quorum quenching studies have also been done on *P*. *aeruginosa* using *Bacillus spp* [22].

Biosensors, liquid chromatography coupled to UV and tandem mass spectrometry (HPLC-UV and LC-MS/MS), gas chromatography coupled to tandem mass spectrometry (GC-MS/MS, MALDI-MS, SFC-MS have all been used to detect and/or quantify N-HLs [6, 23–26]. LC-MS/MS and GC-MS/MS are the most reliable techniques due to the high sensitivity and selectivity that can be achieved using MS/MS [26]. Until recently, the vast majority of methods for detection and quantification of N-HLs could not distinguish between N-HL enantiomers and it was implicitly assumed that all N-HLs had the L-configuration [8, 9]. The chirality of most molecules plays a crucial role in biological systems. Prominent examples have been shown for the effects of D-amino acids on mammalian physiology [27, 28]. The first report of D-N-HLs was that of D-*N*-decanoyl homoserine lactone in *Burkholderia cepacia* [29]. Recently, D-*N*-hexanoyl homoserine lactone in cultures of *Vibrio fischeri* and D-*N*-octanoyl homoserine lactone in cultures of both *B*. *cepacia* and *V*. *fischeri* were extensively evaluated [9]. In this work, the first known examples of D-*N*-3-hydroxyacyl homoserine lactones, and D-*N*-3-oxoacyl homoserine lactones in bacterial supernatants are reported, in addition to the first instance of D-*N*-butanoyl homoserine lactone. The temporal production of a variety of different D-N-HLs in minimal media cultures of *P*. *atrosepticum* and *P*. *aeruginosa* are reported for the first time.

To unify varying nomenclature, Table 1 lists the N-HLs explored in this study and their abbreviations that will be used throughout this work. N-HLs indicate all *N*-acyl homoserine lactones, AHLs represent unsubstituted acyl N-HLs, HHLs represent 3-hydroxy substituted N-HLs and OHLs represent 3-oxo substituted N-HLs. The general notation is D,L-XCY where D,L represents the chirality of the lactone ring, X represents the substitution (A: unsubstituted, H: 3-hydroxyacyl, and O: 3-oxoacyl). Similarly, Y represents the number of carbons on the acyl chain. For HHLs, even though there are two chiral centers, the D,L designation refers to the chirality of the lactone ring. Therefore, there are two L and D diastereomeric pairs respectively.

**Table 1:** Abbreviations of *N*-acyl homoserine lactones used in this study.

Homoserine lactone	Abbreviation
D,L-*N*-acyl homoserine lactone	D,L-N-HL
Unsubstituted N-HLs	D,L-AHL
D,L-*N*-3-oxoacyl homoserine lactone	D,L-OHL
D,L-*N*-3-hydroxyacyl homoserine lactone	D,L-HHL
D,L-*N*-butanoyl	D,L-A-C4
D,L-*N*-hexanoyl	D,L-A-C6
D,L-*N*-octanoyl	D,L-A-C8
D,L-*N*-decanoyl	D,L-A-C10
D,L-*N*-3-oxobutanoyl	D,L-O-C4
D,L-*N*-3-oxohexanoyl	D,L-O-C6
D,L-*N*-3-oxooctanoyl	D,L-O-C8
D,L-*N*-3-oxodecanoyl	D,L-O-C10
D,L-*N*-3-oxododecanoyl	D,L-O-C12
D,L-*N*-3-oxotetradecanoyl	D,L-O-C14
D,L*-N*-3-hydroxybutanoyl	D,L-H-C4
D,L-*N*-3-hydroxyhexanoyl	D,L-H-C6
D,L-*N*-3-hydroxydecanoyl	D,L-H-C10

## Materials and methods

### Chemicals and materials

HPLC grade methanol and acetonitrile, reagent grade dichloromethane, M9 media, D-(+)-glucose, and magnesium sulfate were purchased from MilliporeSigma (St. Louis, MO). A Thermo Scientific^™^ Barnstead^™^ GenPure^™^ Pro water purification system was used to supply deionized (DI) water with 18.2 MΩ-cm resistivity. Racemic standards of O-C4, O-C10, H-C4, H-C6 and H-C12-N-HLs and L-O-C12-N-HL were purchased from Chemodex Ltd. (St. Gallen, Switzerland). Racemic standards of A-C4, A-C6, A-C8, A-C10, A-C12, A-C14, O-C6, O-C8, O-C14, H-C8, H-C10, and H-C14-N-HLs were purchased from MilliporeSigma (St. Louis, MO). ZORBAX SB-C18 (150 mm x 4.6 mm i.d., 5 μm) columns were purchased from Agilent Technologies, Inc. (Santa Clara, CA). ChiralPak^®^ IC-3 (250 mm x 4.6 mm i.d., 3μm) columns were purchased from Chiral Technologies, Inc. (Ann Arbor, MI). β-DEX^™^ 225 (30 m × 0.25 mm, 0.25 μm film thickness) and Supel^™^-Select HLB SPE tubes were provided by MilliporeSigma (Supelco, Bellefonte, PA).

### Sample preparation

Standards were diluted to a concentration of 400 μg/mL in acetonitrile from which 1 μg/mL working solutions were prepared for the calibration curve. M9 media was prepared according to instructions and autoclaved for sterilization. Then, 20% (w/w) glucose and 1M magnesium sulfate were added to the media. For extraction, an SPE manifold coupled to a vacuum pump was used. The SPE cartridges were conditioned with 10 mL each of acetonitrile, methanol, and water respectively. 10 mL of media was spiked with 7.5 μL of a 4 μg/mL internal standard (D,L-A-C7-N-HL) solution and processed through the SPE cartridge. The column was washed with 10 mL of 95:5 methanol: water and eluted with 11 mL of acetonitrile. The samples were then evaporated using a rotatory evaporator and transferred using dichloromethane. Transferred samples were evaporated using a gentle stream of N_2_, and reconstituted with 100 μL of methanol, then transferred to HPLC vials for analysis. For GC-MS/MS, all samples were prepared similarly with the addition of a derivatization step. The samples were first dried using a gentle stream of nitrogen. Then 75 μL of BSTFA with 1% TMCS and 25 μL acetonitrile (co-solvent) was added to the dried sample, mixed for 10 seconds and eventually placed in a 130°C sand bath for 45 minutes.

### Bacterial samples and growth conditions

Samples for *P*. *aeruginosa* ATCC 27853 were obtained from Carolina Biological Supply Company (Burlington, NC). *P*. *atrosepticum* SCRI 1043 samples were purchased from American Type Culture Collection (Manassas, VA). Bacterial samples were spiked into M9 media and allowed to grow in a shaker incubator overnight at 30°C for *P*. *aeruginosa* and 25°C for *P*. *atrosepticum*. From the overnight growth, 200 μL of sample was inoculated into 200 mL of fresh M9 media in triplicate. Inoculated media were grown using a shaker incubator at respective temperatures. 11.5 mL aliquots were taken at different time intervals. From the aliquots, 1.5 mL was collected and diluted with 1.5 mL of water and OD 600 reading were taken using a Vernier SpectroVis^™^ Plus spectrophotometer. The samples were centrifuged for 30 mins and supernatants collected. Supernatants were processed using the SPE method described above and analyzed using LC-MS/MS and GC-MS/MS, for which the conditions are outlined below. Preliminary studies conducted on *P*. *atrosepticum* revealed that the production of D-N-HLs was occurring at times >40 h and even up to ~120 h. Hence, a 124 h growth period was selected. Similar to *P*. *atrosepticum*, preliminary studies of *P*. *aeruginosa* were done to select a growth period of 96 h.

### Chromatographic conditions

#### LC-MS/MS

Shimadzu LC-MS 8040 system (Shimadzu Scientific Instruments, Columbia MD) with electrospray ionization (ESI) and a triple quadrupole mass spectrometer was used in the positive ion mode for the LC-MS/MS analysis. The analysis was done in multiple reaction monitoring (MRM) mode with optimized MRM transitions and collision energies for each enantiomeric standard. The nebulizing gas flow and drying gas flow were 3.0 and 15.0 L/min respectively. The interface voltage was 4.5 kV, and the heat block temperature was 400°C. ZORBAX SB-C18 column was used in tandem with a CHIRALPAK IC-3. A flow rate of 0.4 mL/min was used with a gradient starting at 60:40 methanol (0.1% FA): water (0.1% FA) held for 25 mins, then ramped to 90:10 methanol:water over 12.5 minutes and finally stepped up to 95:5 methanol:water at 37.5 minutes and held there until 65 minutes.

#### GC-MS/MS

Shimadzu GCMS-TQ 8040 (Shimadzu Scientific Instruments, Columbia, MD) was used for GC-MS/MS analysis. It was equipped with a β-DEX^™^ 225 (30 m × 0.25 mm, 0.25 μm film thickness). He was used as a carrier gas with a constant flow of 1.1 mL/min (40 cm/s). The oven temperature was held at 160°C for 10 minutes and increased at a rate of 1°C/min to 230°C and then held for 50 min. The injection mode was splitless with a 1μL injection and 2 min split time with a 20:1 split ratio. The interface temperature was 230°C and the MS ion source temperature was 280°C. Electron ionization (EI) was set to 70 eV. MRM mode was used with optimized collision energies and Q1 and Q3 voltages.

### Analysis of N-HL standards

Racemic standards of D,L-A-C4-C14, D,L-H-C4-C14, and D,L-O-C4-C14 (excluding C-12) were run on LC-MS/MS and GC-MS/MS. Due to the lack of a racemic standard for O-C12 only L-O-C12 was tested. Identification of N-HLs in bacterial samples was done based on matching the retention times, and three characteristic product ions to those of the synthetic standards. LC chromatograms of N-HLs detected in this study are presented in Figs 3 & 4. All enantiomeric pairs had 2 peaks except for HHLs. HHLs had three peaks for majority of the standard solutions: the first peak is a combination of the two L diastereomers, and the second and third peak represent the D counterparts, which are designated as D1 and D2 respectively (Fig 3). For H-C10 and H-C14, there was some resolution between the L diastereomers. There is ample resolution between homologues for quantitation. All enantiomeric pairs are sufficiently resolved except for O-C6. Peak processing software was used to resolve O-C6 through deconvolution methods and subsequently quantify D-O-C6. The resolution of the analytical signals was obtained by iterative curve fitting using exponentially modified Gaussians. Other peaks were observed but eliminated as matrix peaks based on the retention time and mass transitions.

### Quantitation

The calibration curve for LC-MS/MS included concentrations of 5, 12.5, 25, 50, 125, 250, 500, and 1000 ng/mL per enantiomer for all standard N-HLs. The calibration curve for GC-MS/MS included concentrations of 500, 750, 1000, 1500, 2000 ng/mL per enantiomer for all standard N-HLs listed above. Samples for both LC-MS/MS and GC-MS/MS were processed in triplicate using the respective methods outlined above. All quantitation was done using the calibration curves for LC-MS/MS.

## Results and discussion

### Production of D-OHL and D-HHL in *P*. *atrosepticum*

**Fig 2:**
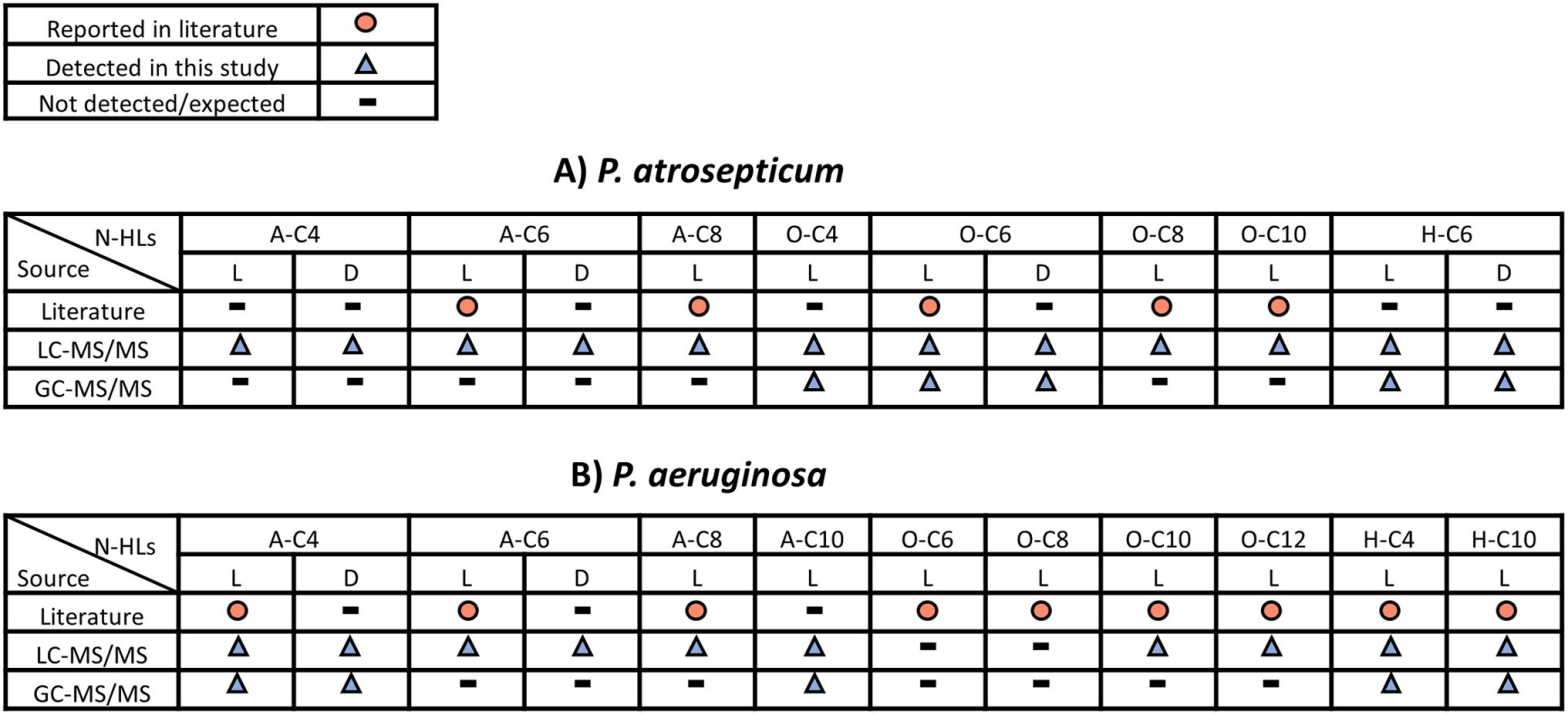
N-HLs detected in by LC-MS/MS and GC-MS/MS compared to N-HLs reported previously in the literature. (A) *P*. *atrosepticum* and (B) *P*. *aeruginosa*. Sources of previously reported N-HLs referenced in Results and Discussions.

**Fig 3:**
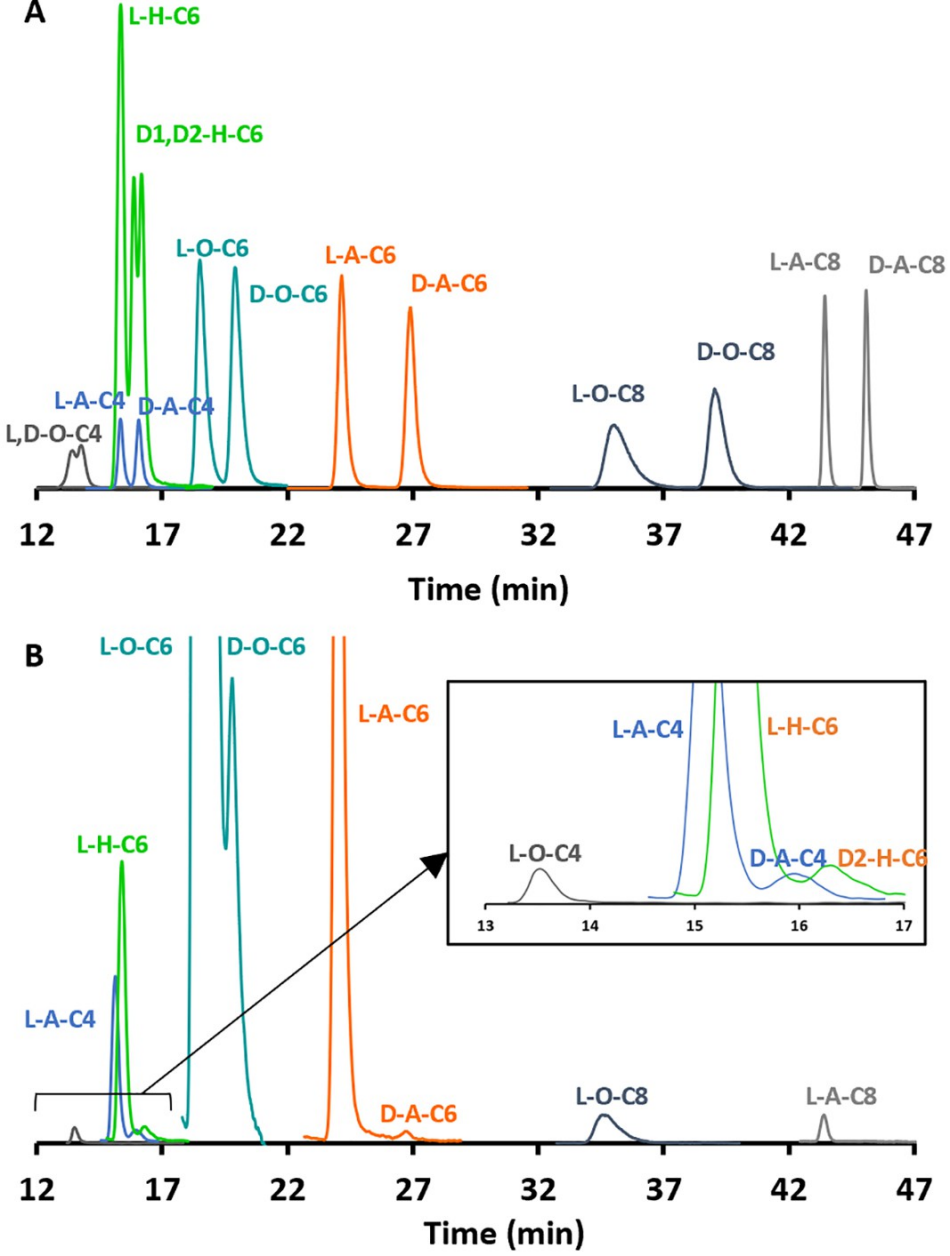
LC-MS/MS chromatogram with MRM transition to m/z = 102 showing the separation of most prominent N-HLs. (A) standard solution and (B) 123 h growth culture of *P*. *atrosepticum*. See Materials and methods section for details. Baselines normalized for clarity.

The large variety of N-HLs produced by *P*. *atrosepticum* during its growth period are summarized in Fig 2A, along with previously reported N-HLs. N-HLs detected via LC-MS/MS versus GC-MS/MS are indicated. *P*. *atrosepticum* produced all classes of N-HLs (AHL, HHL, and OHL) in both enantiomeric forms. The N-HLs detected were D,L-A-C4, D,L-A-C6, D,L-O-C6, and D,L-H-C6. This is the first reported case of D-HHL and D-OHL in any bacterial matrix. Interestingly, A-C8, O-C4, O-C8, and O-C10 were only detected in their L enantiomeric forms. LC-MS/MS chromatograms of standards and bacterial samples for D,L-A-C4, D,L-A-C6, D,L-O-C6, and D,L-H-C6, L-A-C8 and L-O-C8 are presented in Fig 3. H-C6 N-HL (i.e., a hydroxy-substituted N-HL that has two chiral centers) shows three peaks. The first peak is a combination of the L diastereomers, while the second and third peaks correspond to the D-diastereomers and are designated as D1 and D2 respectively (Fig 3). GC-MS/MS was used to confirm the presence of L-O-C4, D,L-O-C6, and D,L-H-C6. Of these N-HLs, L-A-C6, L-A-C8, L-O-C6, L-O-C8, L-O-C10 were expected based on previous studies [10, 21, 30, 31]. However, these N-HLs were reported for a different strain of *P*. *atrosepticum*. Different strains have been shown to produce distinct N-HLs [10, 21, 32]. For SCRI 1043, L-O-C6 has been reported as the major N-HL involved in QS [21, 32]. This study shows that the D-enantiomer also is produced.

### Production of D-AHLs in *P*. *aeruginosa*

**Fig 4:**
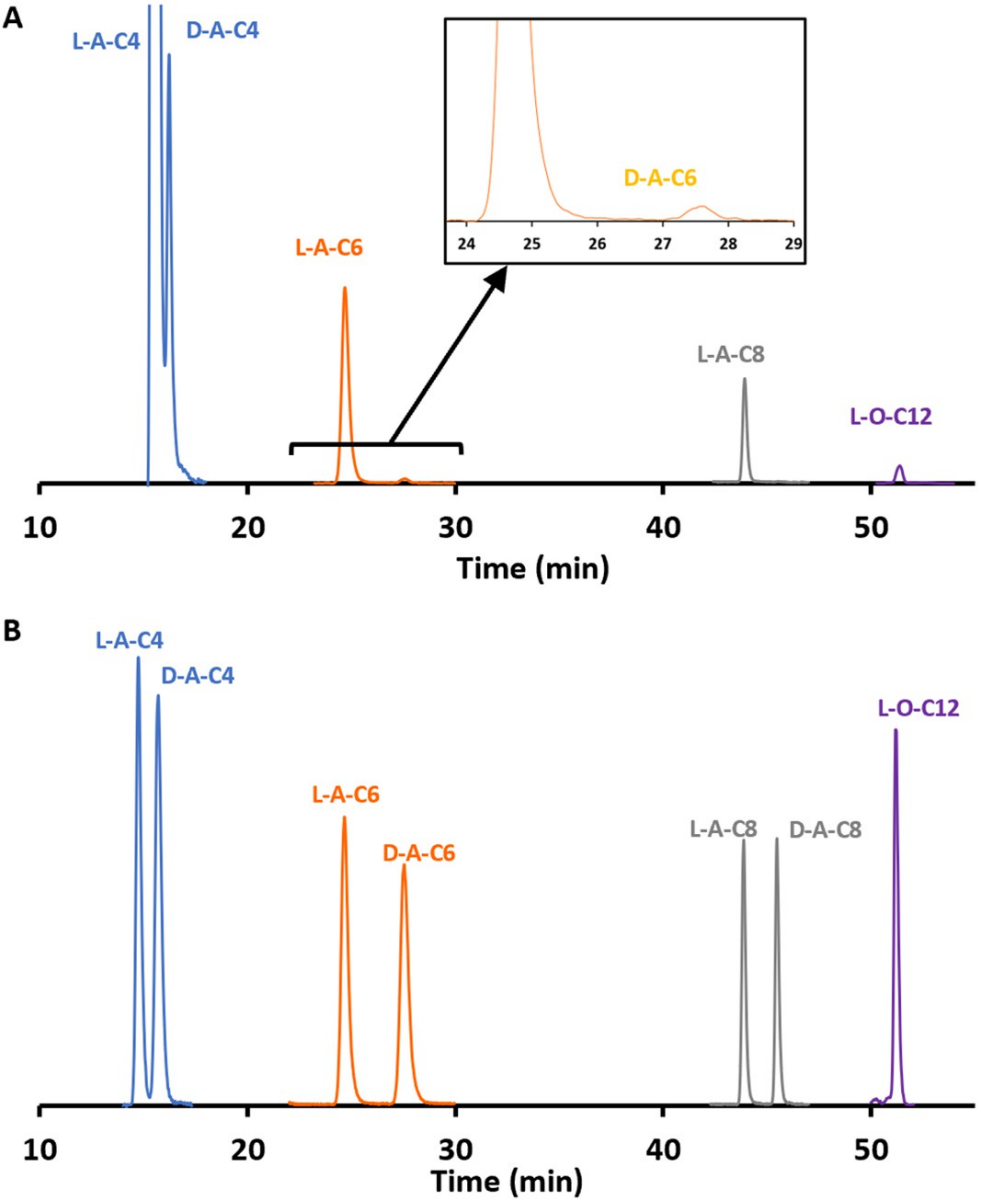
LC-MS/MS chromatogram with MRM transition to m/z = 102 showing the separation of most prominent N-HLs. (A) standard solution and (B) growth culture of *P*. *aeruginosa*. A-C4 and A-C6 were selected from 96 h growth and A-C8 and O-C12 were selected from 36 h growth. See method in experimental. Baselines normalized for clarity.

N-HLs found for *P*. *aeruginosa* in this study are summarized in Fig 2B along with previously reported N-HLs. D,L-A-C4 and D,L-A-C6 were detected for *P*. *aeruginosa*. This is the first report of D-A-C4 in bacterial samples. Only L-enantiomers of A-C8, A-C10, O-C10, O-C12, H-C4, and H-C10 were detected. GC-MS/MS was used to confirm the presence of D,L-A-C4, L-A-C10, L-H-C4, and L-H-C10. LC-MS/MS chromatograms of standards and bacterial samples for D,L-A-C4 and D,L-A-C6, L-A-C8, and L-O-C12 are presented in Fig 4. The expected (from previous reports) N-HLs for *P*. *aeruginosa* (strain PAO1) include the L-enantiomers of A-C4, A-C6, A-C8, O-C6, O-C8, O-C10, O-C12, H-C4, and H-C10 [33, 34]. Of these, O-C6 and O-C8 were not detected in this study. Like *P*. *atrosepticum*, this could be due to a difference in strain or additionally, the variations in growth conditions. In both bacteria, different classes of N-HLs (AHL, HHL, and OHL) with different chain lengths were detected. Unlike *P*. *atrosepticum*, which produced a variety of D-N-HLs, *P*. *aeruginosa* only produced D-NHLs with an acyl side chain (i.e., the AHL variety).

### Enantiomeric production of N-HLs during the growth of *P*. *aeruginosa* and *P*. *atrosepticum*

**Fig 5:**
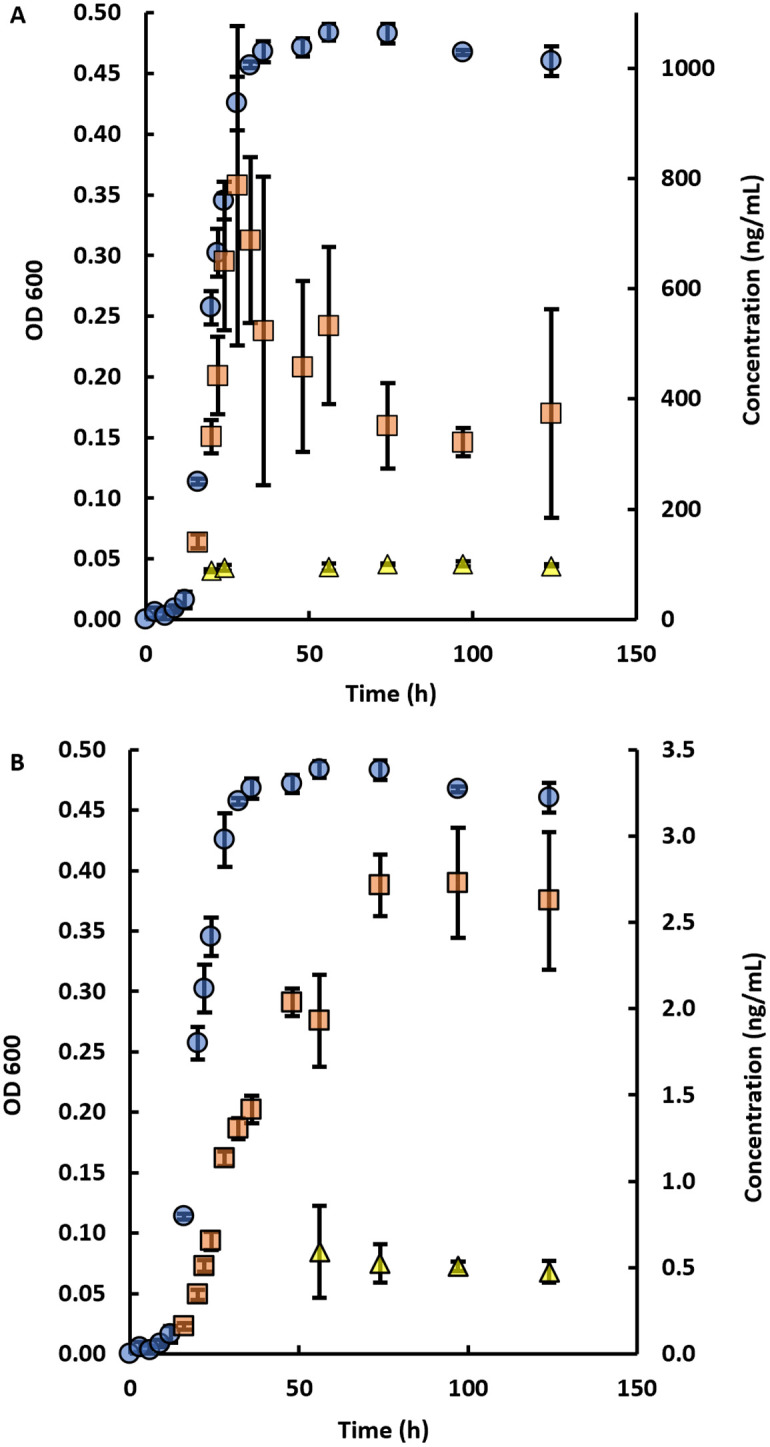
Growth curves showing the OD 600 and production of N-HLs with respect to time in *P*. *atrosepticum*. (A) D,L-O-C6 and (B) D,L-H-C6-HLs. Average of triplicates shown with standard deviations as error bars. Blue circle indicates the OD 600 reading with the measured values in the left y-axis. Red Square indicates the concentration of L-O-C6 (A) and L-H-C6 (B) with measured values in the right y-axis. Yellow triangle indicates the concentration of D-O-C6 (A) and D-H-C6 (B) with measured values in the right y-axis. Concentrations of D-O-C6 was increased by 100 times for clarity.

The bacterial growth curve for *P*. *atrosepticum* is shown in Fig 5, along with the production of two novel N-HLs, i.e., O-C6 and H-C6. For *P*. *atrosepticum*, the exponential growth started at ~12 h, reached a maximum at ~36 h and remained constant over the next ~100 hours (Fig 5). At its maxima, L-O-C6 had a concentration of 800 ± 300 ng/mL and D-O-C6 had a concentration of 1.01 ± 0.04 ng/mL, while L-H-C6 and D-H-C6 had concentrations of 2.7 ± 0.3 and 0.6 ± 0.3 ng/mL respectively. Two distinct production patterns were observed for L-O-C6 and L-H-C6. The concentration of L-O-C6 followed the OD 600 growth curve until ~28 h and declined thereafter (Fig 5A). Detectable amounts of D-O-C6 appeared ~24 h and remained constant until the end of the growth period. These patterns were different for L-H-C6 (Fig 5B), which followed the OD 600 curve more closely. D-H-C6 appeared at ~56 h and remained constant thereafter.

**Fig 6:**
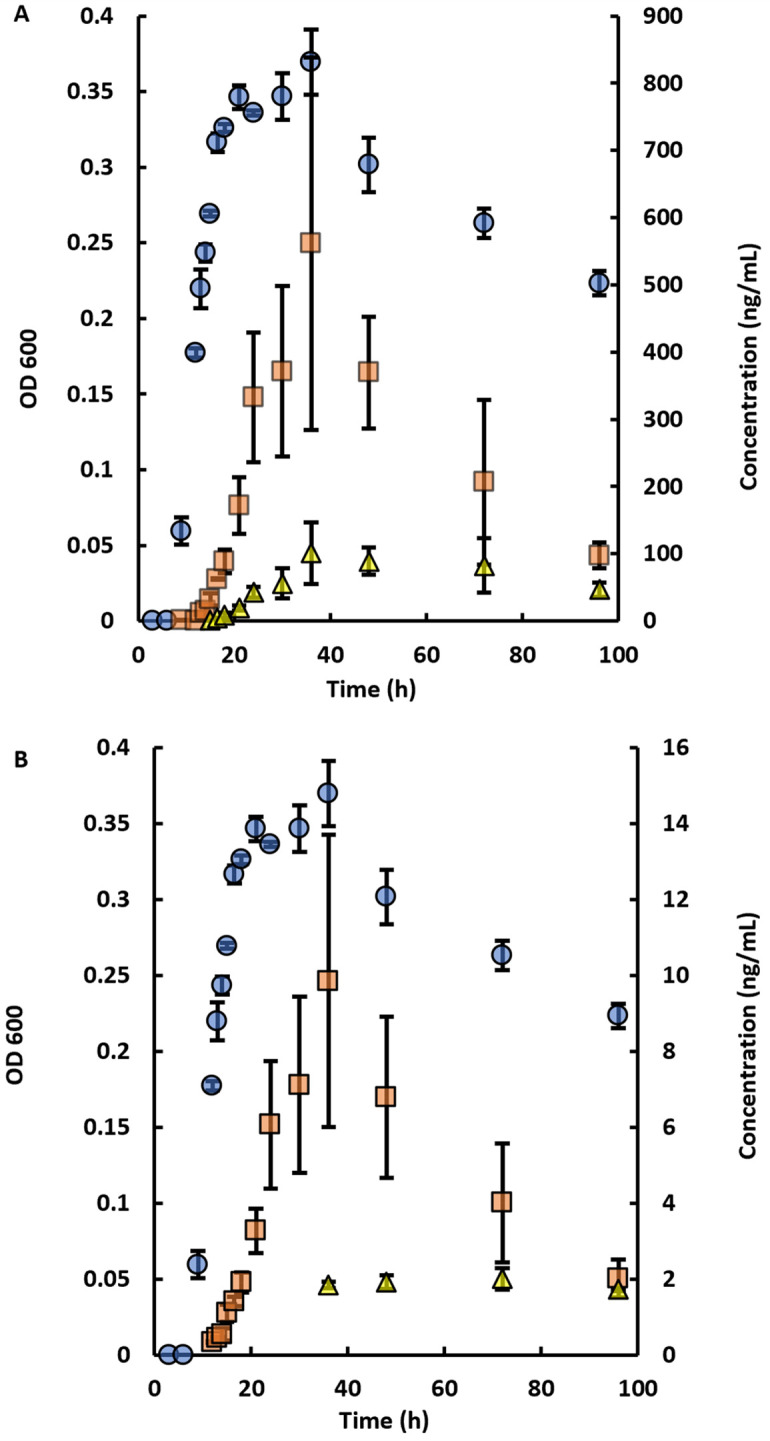
Growth curves showing the OD 600 and production of with respect to time in *P*. *aeruginosa*. (A) D,L-A-C4 and (B) D,L-A-C6-HLs. Average of triplicates shown with standard deviations as error bars. Blue circle indicates the OD 600 reading with the measured values in the left y-axis. Red square indicates the concentration of L-A-C4 (A) and L-A-C6 (B) with measured values in the right y-axis. Yellow triangle indicates the concentration of D-A-C4 (A) and D-A-C6 (B) with measured values in the right y-axis. Concentrations of D-A-C4 and D-A-C6 were increased by 10 times for clarity.

Fig 6 shows the bacterial growth curve of *P*. *aeruginosa* along with the production of its more novel N-HLs. Growth started at ~9 h, reached a maximum at ~25–30 h with the growth decreasing over the next ~65 hours (Fig 6). A-C4 and A-C6 were the most produced N-HLs for *P*. *aeruginosa*. At the maxima, L-A-C4 and D-A-C4 had concentrations of 600±300 and 10±5 ng/mL respectively (Fig 6A). L-A-C6 and D-A-C6 had lower concentrations at 10±4 and 2.0±0.3 ng/mL (Fig 6B). The production of all L-N-HLs in *P*. *aeruginosa* followed the same general trend as the growth curve. D-A-C4 also followed the same general pattern as the L-A-C4, i.e, peaking at ~25–30 h and then declining slowly until the study was stopped at 96 h. However, the D-H-C6 maintained almost the same concentration (~ 2 ng/mL) from ~24–96 h.

### Comparison of concentrations of L and D N-HLs

The maximum concentrations of N-HLs detected in this study are reported in Table 2. For *P*. *atrosepticum*, L-O-C6 was the most produced N-HL, which is consistent with previous findings [21, 30]. Most of the L-N-HLs were produced in greater amounts than the highest levels of D-N-HLs. The case of O-C8 is particularly interesting as it was produced in the third highest amount, but its D-enantiomer was undetectable. L-A-C4 and L-H-C6 were produced in lower quantities than O-C8 and had quantifiable levels of their respective D-enantiomers. Interestingly, of the two D-diastereomers in H-C6, only D2-H-C6 was detected. L-A-C4 was the highest produced N-HL for *P*. *aeruginosa*, which also is consistent with previous studies [33, 34]. Remarkably, D-A-C4 and L-A-C6 were produced in roughly equivalent amounts (both ~10 ng/mL) and were the second most prevalent N-HLs. As can be seen in the last column of Table 2, the L/D ratios appear to correlate with the concentration of the L-enantiomer. N-HLs with lower concentrations of L-enantiomers had relatively greater amounts of their D-enantiomers present.

**Table 2:** Maximum concentrations of each N-HL produced during the growth period quantified using LC-MS/MS.

Bacteria	L-N-HLs	Maximum concentration (ng/mL)	D-N-HLs	Maximum concentration (ng/mL)	L/D
*P*. *atrosepticum*	L-O-C6	800 ± 300	D-O-C6	1.01 ± 0.04	800 ± 300
	L-A-C6	27 ± 1	D-A-C6	0.29 ± 0.03	90 ± 10
	L-O-C8	5.2 ± 0.2	Not detected	-	-
	L-A-C4	3.4 ± 0.2	D-A-C4	0.15 ± 0.04	26 ± 7
	L-H-C6	2.7 ± 0.3	D2-H-C6	0.6 ± 0.3	5 ± 2
	L-A-C8	0.94 ± 0.05	Not detected	-	-
*P*. *aeruginosa*	L-A-C4	600 ± 300	D-A-C4	10 ± 5	60 ± 40
	L-A-C6	10 ± 4	D-A-C6	2.0 ± 0.3	5 ± 2
	L-O-C12	1.8 ± 0.3	Not detected	-	-
	L-A-C8	1.0 ± 0.4	Not detected	-	-
	L-H-C10	0.275 ± 0.002	Not detected	-	-

The growth period for *P*. *atrosepticum* and *P*. *aeruginosa* were 124 h and 96 h respectively. See section Materials and methods for quantitation method.

### Potential roles and production mechanisms of D-N-HLs in bacteria

D-N-HLs have not been considered to be relevant in QS and have not been reported in bacterial systems until recently in *V*. *fischeri* and *B*. *cepacia* [9, 29]. The methodologies used therein were not sufficiently sensitive to detect D-OHLs and D-HHLs. One of the main goals of this work was to examine the occurrence of D-N-HLs in a broader range of bacteria and specifically, to detect D-OHLs and D-HHLs as shown in Figs 3 & 4. In some cases, the concentration of D-N-HLs were as high as 20% of their L-counterparts. The question arises as to the origin of these D-N-HLs as well as their roles in bacterial systems.

*P*. *aeruginosa* has two different QS systems involving LasI and RhlI regulons that are activated by the signaling molecules L-O-C12 and L-A-C4 N-HLs respectively [14]. Both of these QS systems are involved in the production of various virulence factors like exoenzymes alkaline protease, and elastase that induces tissue damage in humans [14]. Interestingly, D-A-C6, which doesn’t have a defined QS system in *P*. *aeruginosa*, was produced in roughly the same concentration as L-O-C12 in this study. Previous investigations on the inhibition and activation of RhlI synthase in *P*. *aeruginosa* have revealed that D-A-C4 inhibits this enzyme [35]. In our study, *P*. *aeruginosa* produced higher concentrations of D-A-C4 than the LasI signaling molecule L-O-C12. In, *P*. *atrosepticum* the highest produced D-N-HL was D-O-C6, which has been shown to activate RhlI [35]. D-O-C6 also has been shown to induce bioluminescence by affecting the LuxR dependent QS system found in *V*. *fischeri* [36]. Both *P*. *atrosepticum* and *P*. *aeruginosa* produced D-A-C6, which has been shown to induce bioluminescence through LuxR as well [36]. Based on these observations, it can be inferred that D-N-HLs can affect the QS systems of their respective L-N-HLs as well as QS systems of other bacteria [35, 36]. A study done on cleavage of D,L-O-C12 by the fatty acid amide hydrolase enzyme revealed that the D-enantiomer was relatively resistant to hydrolysis [37]. A different study involving the hydrolysis of the N-HL lactone ring by a lactonase enzyme showed that D-A-C6 was resistant to hydrolysis compared to its L-counterpart [38]. Thus, it is not unexpected that D-N-HLs accumulate with culture time, whereas L-N-HLs eventually decrease, as shown in this study.

There are at least two pathways for the appearance of D-N-HLs: production through a biosynthetic pathway and post-production racemization [9]. D,L-methionine supplementation studies have been conducted to show that production of D-N-HLs through a biosynthetic pathway, utilizing a D-methionine precursor molecule, is unlikely [9]. In terms of post-production, racemization could occur through a racemizing agent or racemization during the sample preparation procedure, and chromatographic analysis. However, auto-racemization during these processes has been ruled out previously [29, 39]. Racemization due to pH of the complex biological matrix may be possible, however, analysis of supernatant confirmed that the pH change during the chosen growth periods was insignificant. There is a possibility of an enzyme racemase that may convert the L-N-HLs into D-N-HLs. Such racemases are well-known for amino acids [40–42]. In any case, D-N-HLs are found in lower concentrations than their L-counterparts as in the case of most amino acids [27, 43]. Clearly, further investigation is needed in order to understand the origin and role of D-N-HLs in these interesting and important bacterial systems.

## Conclusions

Using comprehensive and sensitive LC-MS/MS and GC-MS/MS methods, the presence of D-N-HLs in growth cultures of *P*. *aeruginosa* and *P*. *atrosepticum* was confirmed. The occurence of unexpected and previously unreported N-HLs was verified for both bacteria. These included novel D-HHLs and D-OHLs which have not been reported previously in any bacteria were. For *P*. *atrosepticum*, D-A-C4, D-A-C6, D-O-C6, and D2-H-C6 were found. For *P*. *aeruginosa*, D-A-C4 and D-A-C6 were first detected. D-A-C4 was detected in high amounts relative to other L-N-HLs in *P*. *aeruginosa*. In general, the concentration of D-N-HLs were usually one to two orders of magnitude lower than their L-counterparts, however, in some cases the ratios were < 10. N-HLs play a role in QS mediated virulence. D-N-HLs have been shown to affect various QS systems. In previous studies, D-A-C4 has been shown to inhibit the L-O-C12 producing QS mechanism involving RhlI while D-O-C6 activated it. D-N-HLs also can be resistant to hydrolysis via fatty acid amide hydrolases and lactonases. Due to these factors, the role of D-N-HLs in bacterial systems should be further explored. If the growing importance of D-amino acids in biological systems is of any indication, the potential role of D-N-HLs in biological systems should be investigated. Of the methods used for analysis, LC-MS/MS was overall more sensitive, and better suited to resolve longer alkyl chain N-HLs. Contrarily, GC-MS/MS gave better resolution of shorter alkyl chain N-HLs, and was used to complement the findings of LC-MS/MS. The origin of D-N-HLs in bacteria is as yet unknown and requires further investigation. As in the case of D-amino acids, a racemase might be involved in the production of D-N-HLs. Likewise, detailed studies on the functions of D-N-HLs in bacterial systems needs to be conducted to explain their potential roles.

## Supporting information

S1 File (DOCX)

## References

[1] Nealson KH, Platt T, Hastings JW. Cellular control of the synthesis and activity of the bacterial luminescent system. J Bacteriol. 1970;104: 313–322. doi: 10.1128/jb.104.1.313-322.1970 5473898 PMC248216

[2] Tomasz A. Control of the competent state in pneumococcus by a hormone-like cell product: An example for a new type of regulatory mechanism in bacteria. Nature. 1965;208: 155–159. doi: 10.1038/208155a0 5884251

[3] Fuqua WC, Winans SC, Greenberg EP. Quorum sensing in bacteria: The LuxR-LuxI family of cell density- responsive transcriptional regulators. Journal of Bacteriology. 1994. doi: 10.1128/jb.176.2.269-275.1994 8288518 PMC205046

[4] Fuqua C, Greenberg EP. Listening in on bacteria: Acyl-homoserine lactone signalling. Nature Reviews Molecular Cell Biology. 2002. doi: 10.1038/nrm907 12209128

[5] Mukherjee S, Bassler BL. Bacterial quorum sensing in complex and dynamically changing environments. Nature Reviews Microbiology 2019 17:6. 2019;17: 371–382. doi: 10.1038/s41579-019-0186-5 30944413 PMC6615036

[6] Morin D, Grasland B, Vallée-Réhel K, Dufau C, Haras D. On-line high-performance liquid chromatography-mass spectrometric detection and quantification of N-acylhomoserine lactones, quorum sensing signal molecules, in the presence of biological matrices. J Chromatogr A. 2003;1002: 79–92. doi: 10.1016/s0021-9673(03)00730-1 12885081

[7] Mohamed NM, Cicirelli EM, Kan J, Chen F, Fuqua C, Hill RT. Diversity and quorum-sensing signal production of Proteobacteria associated with marine sponges. Environ Microbiol. 2008;10. doi: 10.1111/j.1462-2920.2007.01431.x 18211268

[8] Readel E, Portillo A, Talebi M, Armstrong DW. Enantiomeric separation of quorum sensing autoinducer homoserine lactones using GC-MS and LC-MS. Anal Bioanal Chem. 2020;412: 2927–2937. doi: 10.1007/s00216-020-02534-7 32193589

[9] Portillo AE, Readel E, Armstrong DW. Production of both l- and d- N-acyl-homoserine lactones by Burkholderia cepacia and Vibrio fischeri. Microbiologyopen. 2021;10: 1–9. doi: 10.1002/mbo3.1242 34964286 PMC8591449

[10] Latour X, Diallo S, Chevalier S, Morin D, Smadja B, Burini JF, et al. Thermoregulation of N-acyl homoserine lactone-based quorum sensing in the soft rot bacterium Pectobacterium atrosepticum. Appl Environ Microbiol. 2007;73: 4078–4081. doi: 10.1128/AEM.02681-06 17468275 PMC1932719

[11] Smadja B, Latour X, Faure D, Chevalier S, Dessaux Y, Orange N. Involvement of N-acylhomoserine lactones throughout plant infection by Erwinia carotovora subsp. atroseptica (Pectobacterium atrosepticum). Molecular Plant-Microbe Interactions. 2004;17: 1269–1278. doi: 10.1094/MPMI.2004.17.11.1269 15553252

[12] Whitehead NA, Barnard AML, Slater H, Simpson NJL, Salmond GPC. Quorum-sensing in Gram-negative bacteria. FEMS Microbiol Rev. 2001;25. doi: 10.1111/j.1574-6976.2001.tb00583.x 11524130

[13] Burr T, Barnard AML, Corbett MJ, Pemberton CL, Simpson NJL, Salmond GPC. Identification of the central quorum sensing regulator of virulence in the enteric phytopathogen, Erwinia carotovora: The VirR repressor. Mol Microbiol. 2006;59. doi: 10.1111/j.1365-2958.2005.04939.x 16359322

[14] Finch RG, Pritchard DI, Bycroft BW, Williams P, Stewart GSAB. Quorum sensing: A novel target for anti-infective therapy. Journal of Antimicrobial Chemotherapy. 1998;42: 569–571. doi: 10.1093/jac/42.5.569 9848438

[15] Jurado-Martín I, Sainz-Mejías M, McClean S. Pseudomonas aeruginosa: An audacious pathogen with an adaptable arsenal of virulence factors. Int J Mol Sci. 2021;22: 1–37. doi: 10.3390/ijms22063128 33803907 PMC8003266

[16] Chadha J, Harjai K, Chhibber S. Revisiting the virulence hallmarks of Pseudomonas aeruginosa: a chronicle through the perspective of quorum sensing. Environ Microbiol. 2022;24: 2630–2656. doi: 10.1111/1462-2920.15784 34559444

[17] Coutinho H, Falcão-Silva VS, Gonçalves G. Pulmonary bacterial pathogens in cystic fibrosis patients and antibiotic therapy: a tool for the health workers. Int Arch Med. 2008;1: 24. doi: 10.1186/1755-7682-1-24 18992146 PMC2586015

[18] Defez C, Fabbro-Peray P, Bouziges N, Gouby A, Mahamat A, Daurès JP, et al. Risk factors for multidrug-resistant Pseudomonas aeruginosa nosocomial infection. Journal of Hospital Infection. 2004;57. doi: 10.1016/j.jhin.2004.03.022 15236849

[19] Mahmoudi E, Ahmadi A, Sayed-Tabatabaei BE, Ghobadi C, Akhavan A, Hasanzadeh N, et al. A novel AHL-degrading rhizobacterium quenches the virulence of pectobacterium atrosepticum on potato plant. Journal of Plant Pathology. 2011;93.

[20] Crépin A, Barbey C, Cirou A, Tannières M, Orange N, Feuilloley M, et al. Biological control of pathogen communication in the rhizosphere: A novel approach applied to potato soft rot due to Pectobacterium atrosepticum. Plant Soil. 2012;358: 27–37. doi: 10.1007/s11104-011-1030-5

[21] Crépin A, Beury-Cirou A, Barbey C, Farmer C, Hélias V, Burini JF, et al. N-acyl homoserine lactones in diverse Pectobacterium and dickeya plant pathogens: Diversity, abundance, and involvement in virulence. Sensors. 2012;12: 3484–3497. doi: 10.3390/s120303484 22737020 PMC3376598

[22] Musthafa KS, Saroja V, Pandian SK, Ravi AV. Antipathogenic potential of marine Bacillus sp. SS4 on N-acyl-homoserine- lactone-mediated virulence factors production in Pseudomonas aeruginosa (PAO1). J Biosci. 2011;36: 55–67. doi: 10.1007/s12038-011-9011-7 21451248

[23] Cataldi TRI, Bianco G, Palazzo L, Quaranta V. Occurrence of N-acyl-l-homoserine lactones in extracts of some Gram-negative bacteria evaluated by gas chromatography-mass spectrometry. Anal Biochem. 2007;361. doi: 10.1016/j.ab.2006.11.037 17207763

[24] Hoang TPT, Barthélemy M, Lami R, Stien D, Eparvier V, Touboul D. Annotation and quantification of N-acyl homoserine lactones implied in bacterial quorum sensing by supercritical-fluid chromatography coupled with high-resolution mass spectrometry. Anal Bioanal Chem. 2020;412. doi: 10.1007/s00216-019-02265-4 31919609

[25] Yang X, Sun J, Cui F, Ji J, Wang L, Zhang Y, et al. An eco-friendly sensor based on CQD@MIPs for detection of N-acylated homoserine lactones and its 3D printing applications. Talanta. 2020;219. doi: 10.1016/j.talanta.2020.121343 32887072

[26] Horáček O, Portillo AE, Dhaubhadel U, Sung YS, Readel ER, Kučera R, et al. Comprehensive chiral GC-MS/MS and LC-MS/MS methods for identification and determination of N-acyl homoserine lactones. Talanta. 2023;253: 123957. doi: 10.1016/j.talanta.2022.123957 36215752

[27] Armstrong DW, Gasper M, Lee SH, Zukowski J, Ercal N. D-amino acid levels in human physiological fluids. Chirality. 1993;5. doi: 10.1002/chir.530050519 8398594

[28] Armstrong DW, Gasper MP, Lee SH, Ercal N, Zukowski J. Factors controlling the level and determination of D-amino acids in the urine and plasma of laboratory rodents. Amino Acids. 1993;5. doi: 10.1007/BF00805992 24190673

[29] Malik AK, Fekete A, Gebefuegi I, Rothballer M, Schmitt-Kopplin P. Single drop microextraction of homoserine lactones based quorum sensing signal molecules, and the separation of their enantiomers using gas chromatography mass spectrometry in the presence of biological matrices. Microchimica Acta. 2009;166. doi: 10.1007/s00604-009-0183-x

[30] Joshi JR, Khazanov N, Khadka N, Charkowski AO, Burdman S, Carmi N, et al. Direct Binding of Salicylic Acid to Pectobacterium N-Acyl-Homoserine Lactone Synthase. ACS Chem Biol. 2020;15: 1883–1891. doi: 10.1021/acschembio.0c00185 32392032

[31] Barbey C, Crépin A, Bergeau D, Ouchiha A, Mijouin L, Taupin L, et al. In Planta Biocontrol of Pectobacterium atrosepticum by Rhodococcus erythropolis Involves Silencing of Pathogen Communication by the Rhodococcal Gamma-Lactone Catabolic Pathway. PLoS One. 2013;8: 1–9. doi: 10.1371/journal.pone.0066642 23805254 PMC3689677

[32] Chatterjee A, Cui Y, Hasegawa H, Leigh N, Dixit V, Chatterjee AK. Comparative analysis of two classes of quorum-sensing signaling systems that control production of extracellular proteins and secondary metabolites in Erwinia carotovora subspecies. J Bacteriol. 2005;187: 8026–8038. doi: 10.1128/JB.187.23.8026-8038.2005 16291676 PMC1291269

[33] Patel NM, Moore JD, Blackwell HE, Amador-Noguez D. Identification of unanticipatedand novel N-Acyl L-homoserine lactones (AHLs) using a sensitive non-targeted LC-MS/MS method. PLoS One. 2016;11: 1–20. doi: 10.1371/journal.pone.0163469 27706219 PMC5051804

[34] Ortori CA, Dubern JF, Chhabra SR, Cámara M, Hardie K, Williams P, et al. Simultaneous quantitative profiling of N-acyl-l-homoserine lactone and 2-alkyl-4(1H)-quinolone families of quorum-sensing signaling molecules using LC-MS/MS. Anal Bioanal Chem. 2011;399: 839–850. doi: 10.1007/s00216-010-4341-0 21046079

[35] Shin D, Gorgulla C, Boursier ME, Rexrode N, Brown EC, Arthanari H, et al. N-Acyl Homoserine Lactone Analog Modulators of the Pseudomonas aeruginosa Rhll Quorum Sensing Signal Synthase. ACS Chem Biol. 2019;14: 2305–2314. doi: 10.1021/acschembio.9b00671 31545595 PMC6948153

[36] Li SZ, Xu R, Ahmar M, Goux-Henry C, Queneau Y, Soulère L. Influence of the D/L configuration of N-acyl-homoserine lactones (AHLs) and analogues on their Lux-R dependent quorum sensing activity. Bioorg Chem. 2018;77. doi: 10.1016/j.bioorg.2018.01.005 29367078

[37] Palmer AG, Senechal AC, Mukherjee A, Ané JM, Blackwell HE. Plant responses to bacterial N-acyl l-homoserine lactones are dependent on enzymatic degradation to l-homoserine. ACS Chem Biol. 2014;9: 1834–1845. doi: 10.1021/cb500191a 24918118 PMC4136694

[38] Thomas PW, Stone EM, Costello AL, Tierney DL, Fast W. The quorum-quenching lactonase from Bacillus thuringiensis is a metalloprotein. Biochemistry. 2005;44: 7559–7569. doi: 10.1021/bi050050m 15895999

[39] Hodgkinson JT, Galloway WRJD, Casoli M, Keane H, Su X, Salmond GPC, et al. Robust routes for the synthesis of N-acylated-l-homoserine lactone (AHL) quorum sensing molecules with high levels of enantiomeric purity. Tetrahedron Lett. 2011;52: 3291–3294. doi: 10.1016/j.tetlet.2011.04.059

[40] Yoshimura T, Esaki N. Amino acid racemases: Functions and mechanisms. J Biosci Bioeng. 2003;96: 103–109. doi: 10.1016/s1389-1723(03)90111-3 16233494

[41] Radkov AD, Moe LA. Bacterial synthesis of D-amino acids. Appl Microbiol Biotechnol. 2014;98: 5363–5374. doi: 10.1007/s00253-014-5726-3 24752840

[42] Wolosker H, Sheth KN, Takahashi M, Mothet JP, Brady RO, Ferris CD, et al. Purification of serine racemase: Biosynthesis of the neuromodulator d-serine. Proceedings of the National Academy of Sciences. 1999;96: 721–725. doi: 10.1073/pnas.96.2.721 9892700 PMC15203

[43] Armstrong DW, Duncan JD, Lee SH. Evaluation of D-amino acid levels in human urine and in commercial L-amino acid samples. Amino Acids 1991 1:1. 1991;1: 97–106. doi: 10.1007/BF00808096 24194052

