# Supplementary material for: Unusual enantiomeric D,L-N-acyl homoserine lactones in Pectobacterium atrosepticum and Pseudomonas aeruginosa
Source: PLoS One. 2023 Mar 31;18(3):e0283657. doi: 10.1371/journal.pone.0283657 (PMC10065242; doi:10.1371/journal.pone.0283657)
Supplement: S1 File — (DOCX) [file pone.0283657.s001.docx]

**Supplementary Materials**

**Table S1**. SRM transitions monitored when analyzing N-HLs by LC-MS/MS and GC-MS/MS

| N-acyl homoserine lactone | GC-MS/MS*  (m/z>m/z) | LC-MS/MS  (m/z>m/z) |
| --- | --- | --- |
| D,L-A-C4 | 143.00>125.10 | 172.00>102.10 |
| D,L-A-C6 | 143.00>125.10 | 200.05>102.10 |
| D,L-A-C7 | 143.00>125.10 | 214.00>102.10 |
| D,L-A-C8 | 143.00>125.10 | 228.20>102.10 |
| D,L-A-C10 | 143.00>125.10 | 256.25>102.10 |
| D,L-A-C12 | 143.00>125.10 | 284.10>102.10 |
| D,L-A-C14 | 143.00>125.10 | 312.25>102.10 |
| D,L-H-C4 | 244.00>119.10 | 186.00>102.10 |
| D,L-H-C6 | 272.00>200.10 | 216.00>102.10 |
| D,L-H-C8 | 300.10>200.10 | 244.00>102.10 |
| D,L-H-C10 | 328.10>200.10 | 272.10>102.10 |
| D,L-H-C12 | 356.10>200.10 | 300.10>102.10 |
| D,L-H-C14 | 384.10>200.10 | 328.25>102.10 |
| D,L-O-C4 | 157.00>75.10 | 186.00>102.10 |
| D,L-O-C6 | 183.00>75.10 | 214.05>102.10 |
| D,L-O-C8 | 213.00>75.10 | 242.20>102.10 |
| D,L-O-C10 | 241.00>75.10 | 270.10>102.10 |
| L-O-C12 | 269.00>75.10 | 298.10>102.10 |
| D,L-O-C14 | n.a.# | 326.10>102.10 |

* OHL and HHL transitions are for their trimethyl silyl derivatized form.

# D,L-O-C14 was not analyzed by GC-MS/MS

**Table S2.** Comparison of enantiomeric quantification of homoserine lactones using LC-MS/MS and GC-MS/MS. Samples extracted by SPE

| **Analyte** | **LC -MS/MS** | | | **GC – MS/MS** | | |
| --- | --- | --- | --- | --- | --- | --- |
|  | **LOD (ppb)** | **LOQ (ppb)** | **R^2^** | **LOD (ppb)** | **LOQ  (ppb)** | **R^2^** |
| **L** C4-HSL | 4 | 13 | 0.9981 | <LOD | <LOD | <LOD |
| **D** C4-HSL | 2 | 7 | 0.9964 | <LOD | <LOD | <LOD |
| **L** C6-HSL | 2 | 7 | 0.9993 | 382 | 1160 | 0.9730 |
| **D** C6-HSL | 3 | 11 | 0.9992 | 242 | 735 | 0.9890 |
| **L** C8-HSL | 3 | 9 | 0.9994 | 307 | 932 | 0.9824 |
| **D** C8-HSL | 3 | 9 | 0.9998 | 566 | 1716 | 0.9426 |
| **L** C10-HSL | 7 | 23 | 0.9991 | 336 | 1018 | 0.9635 |
| **D** C10-HSL | 8 | 27 | 0.9992 | 880 | 2667 | 0.9425 |
| **L** C12-HSL | 5 | 16 | 0.9995 | 210 | 637 | 0.9854 |
| **D** C12-HSL | 2 | 8 | 0.9918 | 63 | 192 | 0.9987 |
| **L** C14-HSL | 4 | 13 | 0.9979 | 418 | 1268 | 0.9444 |
| **D** C14-HSL | 6 | 19 | 0.9965 | 366 | 1109 | 0.9569 |
|  |  |  |  |  |  |  |
| 3-hydroxy-C4-HSL | Quantitation was not possible because of low extraction recovery. | | | | | |
| **P_1_ + P_2_ ^4^** 3-hydroxy-C6-HSL | 4 | 13 | 0.9972 | 221 | 671 | 0.9838 |
| **P_3_ + P_4_ ^4^** 3-hydroxy-C6-HSL | 5 | 15 | 0.9977 | 209 | 632 | 0.9586 |
| **P_1_ + P_2_** 3-hydroxy-C8-HSL | 5 | 16 | 0.9976 | 123 | 374 | 0.9949 |
| **P_3_ + P_4_** 3-hydroxy-C8-HSL | 5 | 15 | 0.9980 | 3588 | 10872 | 0.8443 |
| **P_1_ + P_2_** 3-hydroxy-C10-HSL | 4 | 14 | 0.9996 | 1296 | 3926 | 0.8833 |
| **P_3_ + P_4_** 3-hydroxy-C10-HSL | 3 | 11 | 0.9989 | 1180 | 3575 | 0.9725 |
| **P_1_ + P_2_** 3-hydroxy-C12-HSL | 4 | 12 | 0.9982 | 83 | 251 | 0.9977 |
| **P_3_ + P_4_** 3-hydroxy-C12-HSL | 5 | 15 | 0.9951 | 535 | 1621 | 0.9744 |
| **P_1_ + P_2_** 3-hydroxy-C14-HSL | 2 | 7 | 0.9908 | 417 | 1264 | 0.9448 |
| **P_3_ + P_4_** 3-hydroxy-C14-HSL | 1 | 5 | 0.9986 | 137 | 414 | 0.9872 |
|  |  |  |  |  |  |  |
| **L** 3-oxo-C4-HSL | Quantitation was not possible because of low extraction recovery. | | | | | |
| **D** 3-oxo-C4-HSL | Quantitation was not possible because of low extraction recovery. | | | | | |
| **L** 3-oxo-C6-HSL | 2 | 8 | 0.9905 | 678 | 2057 | 0.9159 |
| **D** 3-oxo-C6-HSL | 4 | 13 | 0.9900 | 515 | 1561 | 0.9498 |
| **L** 3-oxo-C8-HSL | 2 | 7 | 0.9911 | <LOD | <LOD | <LOD |
| **D** 3-oxo-C8-HSL | 2 | 6 | 0.9905 | <LOD | <LOD | <LOD |
| **L** 3-oxo-C10-HSL | 4 | 13 | 0.9917 | <LOD | <LOD | <LOD |
| **D** 3-oxo-C10-HSL | 2 | 6 | 0.9911 | 446 | 1338 | 0.9625 |
| **L** 3-oxo-C12-HSL | 2 | 7 | 0.9891 | 742 | 2250 | 0.9081 |
| **L** 3-oxo-C14-HSL | 15 | 50 | 0.9917 | n.a. | n.a. | n.a. |
| **D** 3-oxo-C14-HSL | 15 | 51 | 0.9866 | n.a. | n.a. | n.a. |

n.a. = not applicable (this analyte was not analyzed with GC-MS/MS)

^4^ P_1_, P_2_, P_3_, P_4_ stands for first, second, third, and fourth eluted peak of HHLs, respectively
